# Oral contraceptive use by formulation and endometrial cancer risk among women born in 1947–1964: The Nurses’ Health Study II, a prospective cohort study

**DOI:** 10.1007/s10654-020-00705-5

**Published:** 2020-12-17

**Authors:** Norah A. Burchardt, Amy L. Shafrir, Rudolf Kaaks, Shelley S. Tworoger, Renée T. Fortner

**Affiliations:** 1 Division of Cancer Epidemiology, German Cancer Research Center (DKFZ), Heidelberg, Germany; 2 Medical Faculty, Heidelberg University, Heidelberg, Germany; 3 Division of Adolescent/Young Adult Medicine, Department of Pediatrics, Boston Children’s Hospital and Harvard Medical School, Boston, MA USA; 4 Boston Center for Endometriosis, Boston Children’s Hospital and Brigham and Women’s Hospital, Boston, MA USA; 5 Department of Cancer Epidemiology, Moffitt Cancer Center and Research Institute, Tampa, FL USA; 6 Department of Epidemiology, Harvard T.H. Chan School of Public Health, Boston, MA USA

**Keywords:** Oral contraceptives, Hormonal contraceptives, Endometrial cancer

## Abstract

Oral contraceptives (OCs) have been associated with long-term lower endometrial cancer risk; relatively little is known about associations with more recent OC formulations and associations with longer-term risk. A total of 107,069 women from the Nurses’ Health Study II recalled OC use from age 13 to baseline (1989); biennial questionnaires updated data on OC use until 2009. OCs were classified by estrogen and progestin type, dose, and potency based on reported brand. 864 incident endometrial cancer cases were identified through 2017. Multivariable Cox proportional hazards models estimated hazard ratios (HR) and 95% confidence intervals [95% CI] for the association of OC use with endometrial cancer risk. OC use was associated with lower endometrial cancer risk (ever use, HR 0.77 [95% CI 0.65–0.91]; >10 years of use, 0.43 [0.32–0.58] vs. never OC use). Inverse associations for duration were evident regardless of time since last use. Longer durations (> 5 years) of ethinyl estradiol (0.52 [0.41–0.67]) and second-generation progestins (0.43 [0.30–0.61]), both versus never use, were more strongly associated with lower risk than mestranol (0.66 [0.50–0.88], p-het = 0.01) and first-generation progestins (0.62 [0.49–0.78], p-het = 0.03). Inverse associations were generally observed for cross-classified cumulative average estrogen and progestin dose and potency (< vs. ≥ median; ever use vs. never OC use), with the exception of high estrogen and low progestin dose. OCs were associated with lower endometrial cancer risk, independent of time since last use. Use of ethinyl estradiol and second-generation progestins were more strongly inversely associated with risk compared with older formulations.

**Supplementary Information:**

The online version contains supplementary material available at 10.1007/s10654-020-00705-5.

## Introduction

Oral contraceptives (OCs) are among the birth control methods most commonly used by women of reproductive age [1–3]. An estimated 79% of women ages 15–44 years were ever OC users in the United States in 2011–2013 (~ 42.5 million women) [1]. OCs are generally constituted of an estrogen and a progestin; progestin-only OCs are also available, but less frequently prescribed (~ 2% of current OC users in the U.S. in 2006–2010) [4]. The formulation of OCs has changed since their first introduction, with changes in the estrogen component and doses decreasing from 150 µg in the 1960s to as low as 20 µg in modern formulations [2, 5–8]. Four generations of progestins have been utilized, with differing androgenic and metabolic effects [2, 7–10].

Sex steroids have established effects on the endometrium, with estrogen promoting, and progesterone inhibiting, proliferation [11–13]. As described in a meta-analysis and other prospective studies with long-term follow-up [14–18], OC use is associated with consistent reductions (~ 30–40%) in endometrial cancer risk. Previous studies predominantly included women from older birth cohorts, who were likely exposed to OCs containing earlier estrogen and progestin types relative to more contemporary OCs. A recent publication from the Danish Sex Hormone Register Study on the use of contemporary combined hormonal contraceptives and endometrial cancer reported risk reductions with current and recent OC use [19]. While this study provided data on newer OC formulations, it was restricted to women younger than age 50, a population that typically has relatively low incidence of the disease [20], which may limit the generalizability of these findings. To our knowledge, no prior prospective study has provided a detailed evaluation of longer-term endometrial cancer risk by OC formulation. Thus, we evaluated the associations between OCs and endometrial cancer risk by estrogen and progestin types (mestranol, ethinyl estradiol, first- and second-generation progestins), in a prospective study of women born from 1947 to 1964.

## Methods

### Study population

The Nurses’ Health Study II (NHSII) is a prospective cohort study initiated in 1989 when registered nurses from 14 states of the U.S. completed and returned a baseline questionnaire covering a broad range of lifestyle, health, and reproductive factors. The cohort includes 116,429 female nurses aged 25–42 years at recruitment, with follow-up for health outcomes and updates to lifestyle and risk-factor data via biennial mailed questionnaires and has been described in detail previously [21, 22]. The study was approved by the institutional review boards of the Brigham and Women’s Hospital and Harvard T.H. Chan School of Public Health, and those of participating registries as required.

### Oral contraceptive use

To determine the use of OCs of each participant before baseline, a life-events calendar was created from the age of 13. Women reported if they used OCs, for how many months, and from which brand, for each year of age. Reported OC use for 2 months or more but less than 10 months in a year was counted as 6 months of use in that year, 10 or more months of use in a year was counted as 12 months, and less than 2 months of use in a year was counted as no use. In the follow-up questionnaires up until 2009 participants reported whether they were currently using OCs, if they had used OCs during the last 2 years, the duration of use during this period, and the brand used the longest. A booklet with names and color photographs of all OC brands available during the relevant period was provided to aid participant recall. Only premenopausal OC use was considered for this study.

#### OC type, dose, and potency

OCs were classified by estrogen and progestin types, doses, and potencies [23]. Two estrogen types [mestranol (ME) and ethinyl estradiol (EE)], and four generations of progestin [first-(P1), second-(P2), third-(P3), and fourth-generation (P4)], were used for categorization [24]. First-generation progestins (P1) include norethindrone, norethynodrel, norethindrone acetate, ethynodiol diacetate (estranes derived from testosterone), medroxyprogesterone acetate, and chlormadinone acetate (pregnanes derived from 17-OH-progesterone), second-generation progestins (P2) include levonorgestrel and norgestrel (gonanes derived from testosterone), third-generation progestins (P3) include desogestrel and norgestimate (gonane derivatives), and fourth-generation progestins (P4) include drospirenone (non-ethylated estrane).

Estrogen and progestin doses were determined according to the reported brand and formulation of OC. Estrogen potencies, expressed in micrograms (µg) of ethinyl estradiol (EE) equivalents per day, were determined according to mouse uterine assays, while progestin potencies, expressed in milligrams (mg) of norethindrone equivalents per day, were determined through the induction of glycogen vacuoles in human endometrium [25–27].

### Covariate data collection

All variables were assessed on the baseline and follow-up questionnaires as detailed below. Age was calculated using birthdate and questionnaire return date. Age at menarche and height were reported at baseline. Other covariates used for adjustments included (year of data collection): weight (biennially); menopausal status and age at menopause (biennially); postmenopausal hormone therapy use (HT; biennially); number of full-term pregnancies (biennially until 2009); tubal ligation, use of intrauterine device (IUD), diaphragm, or other types of contraception (biennially until 2009); smoking status (biennially; at baseline in 1989 women who smoked < 20 packs of cigarettes during their lifetime and were not current smokers were classified as never smokers); laparoscopically confirmed endometriosis (biennially from 1993 to 2013); and diagnosis of polycystic ovary syndrome (PCOS; in 1989 and biennially from 1993 to 2001). Body mass index (BMI, kg/m^2^) was calculated using weight and height.

### Endometrial cancer cases

A total of 1491 endometrial cancer cases were self-reported on biennial questionnaires through 2017. Medical records were requested for all cases; pathology information was accessed through state cancer registries when not available. Information on deceased women were obtained from the National Death Index, the U.S. Postal Service, or next of kin. Medical records from 58% (n = 862) of all self-reported cases were received and reviewed by study physicians; 91% (n = 784) were confirmed. Known non-epithelial cases were excluded (n = 56). Given the high confirmation rate, participant-reported cases confirmed on re-contact were included (n = 208), resulting in 936 cases before further exclusions.

Histological subtype, grade, and stage at diagnosis were obtained from pathology records. Cases were classified as type I/endometrioid or type II/non-endometrioid (clear cell adenocarcinoma, squamous cell carcinoma, and adenosquamous carcinoma) and as invasive or non-invasive (i.e., no myometrial invasion). Study physicians were blinded to OC use status.

### Statistical analyses

After exclusions for missing birthdate (n = 17), prevalent endometrial cancer (n = 1), prior cancer (except non-melanoma skin neoplasm; n = 1039), prior hysterectomy (n = 6196), and missing duration of OC use (n = 2107), 107,069 women including 864 endometrial cancer cases remained in the analysis.

Person-years were calculated from baseline to the date of endometrial or other cancer diagnosis (except non-melanoma skin neoplasms), hysterectomy, death, or end of study follow-up (June 2017), whichever occurred first. We used multivariable Cox proportional hazards models with time-varying covariates stratified by age in months and calendar period to calculate hazard ratios (HR) and 95% confidence intervals (CI). Covariates were selected a priori and included BMI (kg/m^2^, continuous), number of full-term pregnancies (continuous), smoking status (never/past/current), menopausal status (premenopausal/postmenopausal/perimenopausal or unknown), use of HT (never/ever), diagnosis of PCOS (yes/no), laparoscopically confirmed endometriosis (yes/no), age of menarche (years), and use of IUD/diaphragm (yes/no). Further adjustment for diagnosis of diabetes mellitus and hypertension did not change the risk estimates substantially and were therefore not included in the final models. There were no violations of the assumption of proportional hazards; this was tested by evaluating interaction by time in our models.

#### Missing data

Women missing OC use data in a given biennial questionnaire follow-up cycle did not contribute to the analysis for current use for that cycle (i.e., did not contribute person-time); data for ever use and duration of use were carried forward for one cycle in premenopausal women, and perpetually for postmenopausal women. Missing data was limited for covariates. Highest frequency was observed for BMI with < 11% missing in any given follow-up cycle; data were carried forward from the previous cycle for one cycle if missing, resulting in < 4% missing in any given cycle. The proportion of missing data was limited for all other covariates (overall ≤ 5.8%). There were no clear patterns in missing values evident across exposure categories. A missing indicator was used for categorical variables, and for continuous variables simple imputation using the median value for the whole cohort in the same follow-up cycle was used, together with a missing indicator variable.

#### Duration of OC use and time since last OC use

Duration of OC use was categorized as never, ≤ 1 year, > 1–5 years, > 5–10 years, and > 10 years of use. Time since last OC use was classified as never use, current use, ≤ 5 years, > 5–10 years, > 10–15 years, and > 15 years since last use. We also examined the associations with duration of OC use and time since last use continuously (per year). Cross-classification of duration of OC use and time since last use was categorized in never use, ≤ 1 year of use, > 1–5 years of use/≤ 10 years since last use, > 1–5 years of use/>10 years since last use, > 5 years of use/≤10 years since last use, and > 5 years of use/>10 years since last use. We further assessed duration of and time since last use by analyzing continuous duration of OC use by categorical time since last use and vice versa (e.g., continuous time since last use in categories of duration of use (≤ 1, > 1–5, > 5–10, and > 10 years)). P for trend was modelled using the exposures as ordinal variables with values set to the category medians.

#### Duration of OC use by estrogen and progestin types

Duration of use of the two different types of estrogen (ME and EE) and the four generations of progestins (P1, P2, P3, and P4) was classified in categories (never OC use, ≤ 1 year, > 1–5 years, and > 5 years) and evaluated continuously (per year). These analyses were not restricted to exclusive users of each type of estrogen or progestin (i.e., if a participant used more than one type of estrogen and/or progestin, she was included in each of the relevant analyses [e.g., ME and EE]; however, exclusive users of one type of estrogen or progestin were excluded from the analyses of duration of use of the other type(s) of estrogen or progestin.

#### Cumulative and average dose and potency

Cumulative doses and potencies of estrogen and progestin for each participant were calculated using the duration of use of each OC formulation. Due to the correlation of these variables with total duration of OC use, we evaluated cumulative dose and potency residuals. The residuals correspond to the difference between a participant’s predicted cumulative dose/potency and the observed value. The residuals were calculated through log-transformation of the cumulative dose and potency variables, which were then regressed on the natural log of total OC duration. Analyses of the residuals were adjusted for OC use duration.

Cumulative averages were obtained by dividing the cumulative doses and potencies of estrogen and progestin by the total duration of OC use in months. The median values observed among OC users were used to categorize the average doses and potencies (never users, < median, ≥ median). We additionally cross-classified participants by cumulative average dose and potency of both estrogen and progestin (e.g., never use; dose of estrogen/progestin < median/< median, < median/≥ median, ≥ m edian/< median, and ≥ median/≥ median; potency of estrogen/progestin < median/< median, < median/≥ median, ≥ median/< median, and ≥ median/≥ median). Median cutpoints were defined per follow-up questionnaire cycle and ranged from 1081.01 to 1134.36 µg per month for estrogen dose, 20.26–21.56 mg per month for progestin dose, 776.15–801.05 µg EE equivalents per month for estrogen potency; median progestin potency was equal to 22.82 mg norethindrone equivalents per month in all follow-up cycles.

#### Subgroup and sensitivity analyses

We evaluated potential differences in the associations between ever OC use and duration of use and endometrial cancer risk by age (≤ 50 vs. > 50 years), BMI (< 25 vs. 25–< 30 vs. ≥ 30 kg/m^2^), menopausal status (premenopausal vs. postmenopausal), HT use (never vs. ever), and smoking status (never vs. past/current) in stratified analyses; heterogeneity was assessed by inclusion of an interaction term and using likelihood ratio tests (LRTs). We assessed heterogeneity in the associations for invasive and non-invasive endometrial cancer, using LRTs comparing models assuming different versus common associations by subtype [28]. Cochran´s Q-test was used to examine heterogeneity in associations by estrogen and progestin types [29].

We examined potential deviations from linearity of the associations for exposures modelled continuously non-parametrically using restricted cubic splines [30]; LRTs were used to compare models with the linear term to models with both the linear and the cubic spline terms.

To limit reverse causation, we conducted analyses in which we stopped updating OC use 2 years prior to the diagnosis of endometrial cancer or the end of follow-up, whichever occurred first (i.e., 2-year lag). We conducted a further analysis restricted to cases confirmed by medical record review.

Analyses were conducted in SAS 9.4 (SAS Institute Inc., Cary, NC, USA). *P* values were considered statistically significant at < 0.05; all statistical tests were two-sided.

## Results

### Study population

More than 99% of all participants were premenopausal at baseline. Eighty-three percent of all women were ever OC users; 71% of these women reported using OCs for > 1–10 years. (Table 1). Baseline characteristics of ever P1 and P2 users were similar (supplementary table S1). Compared to ever ME users at baseline, a lower proportion of ever EE users were parous (69% vs. 77%) and reported tubal ligation (15% vs. 21%); these differences were attenuated by the end of follow-up (e.g., parous, 83% vs. 85%; supplementaty Table S2). Maximum follow-up time was 27.8 years (mean: 12.7 years).

**Table 1:** Age-adjusted characteristics of the study population by duration of oral contraceptive (OC) use at study baseline in 1989: Nurses’ Health Study II (n = 107,069)

	Duration of OC use, categorical				
	Never use (n = 18,636)	≤ 1 year (n = 17,987)	> 1–5 years (n = 39,180)	> 5–10 years (n = 23,817)	> 10 years (n = 7,449)
Age, years*	34.0 (24.0–44.0)	35.0 (24.0–44.0)	34.0 (24.0–44.0)	34.0 (24.0–44.0)	35.0 (25.0–43.0)
Age at menarche, years	12.0 (9.0–17.0)	12.0 (9.0–17.0)	12.0 (9.0–17.0)	12.0 (9.0–17.0)	12.0 (9.0–17.0)
BMI^a^, categorical					
BMI < 25 kg/m^2^, %	68.0	70.7	70.6	72.0	72.5
BMI 25-<30 kg/m^2^, %	18.6	18.2	18.2	18.3	17.9
BMI ≥ 30 kg/m^2^, %	13.4	11.1	11.1	9.7	9.6
Ever parous, %	60.6	74.8	75.4	71.0	58.1
Number of full-term pregnancies (among parous women)	2.0 (1.0–10.0)	2.0 (1.0–15.0)	2.0 (1.0–9.0)	2.0 (1.0–8.0)	2.0 (1.0–6.0)
Breastfeeding duration, in months (among parous women who ever breastfed)	11.5 (1.5–95.0)	10.0 (1.5–91.0)	10.0 (1.5–95.0)	9.0 (1.5–76.0)	9.0 (1.5–75.0)
Menopausal status					
Premenopausal, %	99.5	99.5	99.6	99.7	99.6
Postmenopausal, %	13.4	11.1	11.1	9.7	9.6
Ever use of other contraceptive methods					
IUD^b^ or diaphragm, %	18.1	16.2	13.6	10.4	7.0
Male contraception, %	32.7	34.2	32.7	28.0	21.9
Tubal ligation, %	9.8	16.4	18.2	17.5	13.1
Other, %	23.6	18.9	15.7	13.1	11.4
Diagnosis of endometriosis^c^, %	2.1	3.6	3.5	3.2	3.1
Diagnosis of PCOS^d^, %	3.8	6.4	5.7	5.1	4.8
Postmenopausal HT^e^ use, %	0.2	0.2	0.2	0.1	0.2
Arterial hypertension, %	5.2	5.3	5.3	4.8	5.4
Diabetes mellitus, %	1.3	0.8	0.7	0.5	0.6
Smoking status					
Never smoker, %	77.2	67.5	65.5	58.7	54.8
Past smoker, %	14.1	20.4	21.8	25.3	25.6
Current smoker, %	8.7	12.1	12.7	16.0	19.6

Values are medians and ranges or percentages and are standardized to the age distribution of the study population

Values of polytomous variables may not sum to 100% due to rounding

*Value is not age adjusted

^a^Body mass index

^b^Intrauterine device

^c^Laparoscopically confirmed endometriosis

^d^Polycystic ovary syndrome

^e^Hormone therapy

### OC use characteristics

Among ever OC users in 1989, 49% had used only 1 brand, 26% had used 2 brands, and 12.5% used 3 or more (13.3% unknown). Ever use of formulations containing EE (66%) and P1 (72%) were more frequently reported than ME (43%) and P2 (37%). P3 and P4 were introduced after the study baseline, therefore none of the participants reported use in 1989. The median average dose was 1,000 µg per month for estrogen (range 23–3000 µg) and 21 mg per month for progestin (range 2–225 mg), while the median average potency was 795.7 µg EE equivalents per month for estrogen (range 23–9999 µg) and 23 mg norethindrone equivalents per month for progestin (range 5–225 mg) (supplementary table S3). Among ever ME users, 100% were ever P1 users and 29% were ever P2 users, while among ever EE users, 76% were ever P1 and 57% were ever P2 users (supplementary table S4).

### Characteristics of the cancer cases

Median age at endometrial cancer diagnosis was 59 years (range 39–69 years). Among the cases with known tumor histology (n = 655), 94% were type I/endometrioid carcinomas, and 64% of these were well-differentiated. Among the cases with known invasiveness (n = 385), 33% were non-invasive carcinomas (Table 2).

**Table 2:** Characteristics of the endometrial cancer cases identified in the Nurses’ Health Study II (1989–2017)

	Endometrial cancer cases (n = 864) n (%)
Age at diagnosis (median [range])	59.0 [39.0–69.0]
Types of epithelial carcinoma	
Endometrioid carcinoma—type I, %	614 (93.7)
Non-endometrioid carcinoma—type II, %	41 (6.3)
*(Missing type, n = 209)*	
Histological subtype	
Adenocarcinoma‚ endometrioid type	614 (94.6)
Serous cell carcinoma	13 (2.0)
Clear cell carcinoma	6 (0.9)
Squamous cell carcinoma	1 (0.1)
Adenosquamous cell carcinoma (mucoepidermoid)	1 (0.1)
Endometrial carcinosarcoma	6 (0.9)
Mixed carcinoma	8 (1.2)
*(Missing subtype, n = 215)*	
Differentiation/grade in endometrioid type	
Well differentiated/G1	389 (63.8)
Moderately differentiated/G2	161 (26.4)
Poorly differentiated/G3	60 (9.8)
*(Missing grade, n = 254)*	
Invasiveness	
Non-invasive carcinoma	129 (33.5)
Invasive carcinoma	256 (66.5)
*(Missing invasiveness, n = 479)*	

Percentages were calculated among the total of cases with available pathology data

Values of polytomous variables may not sum to 100% due to rounding

### Ever OC use, duration of use, and time since last use

Relative to never users, ever OC users had lower endometrial cancer risk (HR 0.77 [95% CI 0.65–0.91]; Table 3), with lower risk associated with longer duration of use (e.g., > 10 years, 0.43 [0.32–0.58]; *p* trend < 0.0001; per year, among ever users: 0.94 [0.92–0.96]). Lower risk was observed among women with > 5 years of OC use, versus never use, with a somewhat stronger association for more recent users (time since last use: ≤ 10 years, 0.43 [0.31–0.60]; > 10 years, 0.62 [0.50–0.78]; Table 3); no association was observed for either category of time since last use among women with shorter-term use. Considering continuous duration of OC use (in years) by categories of time since last use, and continuous time since last OC use (in years) by categories of duration, duration was significantly inversely associated with risk independent of time since last use (HRs per 1 year of use range = 0.90–0.95), while time since last OC use was not associated with risk in any of the duration categories (HRs per 1 year since last use range = 0.99–1.02; supplementary table S5).

**Table 3:** Associations between ever OC use, duration of OC use, and time since last OC use and endometrial cancer risk in the Nurses’ Health Study II (1989–2017)

	Person-years	Cases	Adjusted for age and calendar period		Multivariable-adjusted^a^	
			HR	95% CI	HR	95% CI
*Use of OCs*						
Never use	344,113	175	1.00	(ref.)	1.00	(ref.)
Ever use	1,957,361	689	0.67	(0.56–0.79)	0.77	(0.65–0.91)
Current use	181,298	18	0.53	(0.32–0.87)	0.60	(0.36–0.99)
Past use	1,762,550	663	0.67	(0.57–0.79)	0.77	(0.65–0.92)
*Duration of OC use*						
Never use	344,113	175	1.00	(ref.)	1.00	(ref.)
≤ 1 year	334,666	154	0.82	(0.66–1.02)	0.97	(0.77–1.20)
> 1–5 years	783,484	327	0.78	(0.64–0.93)	0.91	(0.75–1.09)
> 5–10 years	529,236	145	0.55	(0.44–0.68)	0.63	(0.50–0.79)
> 10 years	309,975	63	0.39	(0.29–0.52)	0.43	(0.32–0.58)
Continuous duration of use, in years^b^			0.94	(0.92–0.95)	0.94	(0.92–0.96)
*p*-trend^c^				< 0.0001		< 0.0001
*p*-trend^b^				< 0.0001		< 0.0001
Duration of OC use and time since last OC use^d^						
Never use	344,113	175	1.00	(ref.)	1.00	(ref.)
≤ 1 year of use	334,666	154	0.82	(0.66–1.02)	0.96	(0.77–1.20)
> 1–5 years of use and ≤ 10 years since last use	197,255	40	0.76	(0.53–1.08)	0.89	(0.63–1.27)
> 1–5 years of use and > 10 years since last use	534,797	278	0.79	(0.65–0.95)	0.91	(0.75–1.11)
> 5 years of use and ≤ 10 years since last use	324,053	43	0.37	(0.26–0.52)	0.43	(0.31–0.60)
> 5 years of use and > 10 years since last use	344,061	146	0.55	(0.44–0.69)	0.62	(0.50–0.78)

^a^Adjusted for age (months), calendar period, BMI (kg/m^2^, continuous), number of full-term pregnancies (continuous), smoking status (never/past/current), menopausal status (premenopausal/postmenopausal/perimenopausal or unknown), use of HT (never/ever), diagnosis of PCOS (yes/no), laparoscopically confirmed endometriosis (yes/no), age of menarche (years), and use of IUD/diaphragm (yes/no)

^b^Calculation including ever OC users only

^c^Calculation including never OC users

^d^Current OC users were excluded from this analysis

### Estrogen and progestin type, dose, and potency

Relative to never OC use, longer duration of EE use was more strongly associated with lower endometrial cancer risk than ME use (*p*-het_MEvsEE_ = 0.01; > 5 years ME 0.66 [0.50–0.88], EE 0.52 [0.41–0.67]; Table 4), with similar heterogeneity between P1 and P2 (*p*-het_P1vsP2_= 0.03; > 5 years P1 0.62 [0.49–0.78], P2 0.43 [0.30–0.61]). Median duration of use in the > 5 years categories was 7.2 years for ME, 8.3 years for EE, and 8 years for both P1 and P2. Associations were generally similar among exclusive users of ME, EE, P1, and P2; however, there was a relatively low proportion of exclusive users (ME, 18%; EE, 43%; P1, 44%; P2, 15% of ever OC users by the end of follow-up). Results from mutually adjusted models (e.g., duration of ME use adjusted for duration of EE use, duration of P1 for duration of P2) were comparable for duration of ME, EE, P1, and P2 (Table 4). P3 and P4 use could not be assessed due to the limited numbers of users (ever P3 use, n = 4117; ever P4 use, n = 701 by the end of follow-up).

**Table 4:** Multivariable-adjusted^a^ hazard ratios (HR) and 95% confidence intervals for endometrial cancer in relation to OC use by estrogen and progestin type in the Nurses’ Health Study II (1989–2017)

	Person-years	Cases	HR	95% CI
*Estrogen type*				
Mestranol (ME)				
Never OC use	344,113	175	1.00	(ref.)
Ever ME use	805,651	317	0.74	(0.61–0.90)
Exclusive ME use	374,797	167	0.76	(0.61–0.94)
Duration of ME use				
≤ 1 year	207,686	83	0.79	(0.60–1.03)
> 1–5 years	383,169	155	0.77	(0.62–0.96)
> 5 years	183,683	69	0.66	(0.50–0.88)
Continuous, in years^b^			0.96	(0.92–1.00)
Continuous, in years, adjusted for continuous duration of EE^b^			0.96	(0.92–1.00)
*p*-trend^c^				0.01
*p*-trend^b^				0.22
Ethinyl estradiol (EE)				
Never OC use	344,113	175	1.00	(ref.)
Ever EE use	1,353,870	424	0.76	(0.63–0.91)
Exclusive EE use	855,881	256	0.77	(0.63–0.94)
Duration of EE use				
≤ 1 year	251,449	120	1.08	(0.85–1.37)
> 1–5 years	538,573	177	0.79	(0.64–0.98)
> 5 years	465,600	99	0.52	(0.41–0.67)
Continuous, in years^b^			0.92	(0.90–0.95)
Continuous, in years, adjusted for continuous duration of ME^b^			0.92	(0.90–0.95)
*p*-trend^c^				< 0.0001
*p*-trend^b^				< 0.0001
*p*-het ME versus EE^d^				0.01
*Progestin type*				
First-generation progestin (P1)				
Never OC use	344,113	175	1.00	(ref.)
Ever P1 use	1,409,364	479	0.76	(0.64–0.91)
Exclusive P1 use	905,756	331	0.78	(0.65–0.94)
Duration of P1 use				
≤ 1 year	288,175	126	0.95	(0.75–1.20)
> 1–5 years	589,882	205	0.78	(0.63–0.96)
> 5 years	444,438	124	0.62	(0.49–0.78)
Continuous, in years^b^			0.95	(0.93–0.97)
Continuous, in years, adjusted for continuous duration of P2^b^			0.95	(0.92–0.97)
*p*-trend^c^				< 0.0001
*p*-trend^b^				0.0006
Second-generation progestin (P2)				
Never OC use	344,113	175	1.00	(ref.)
Ever P2 use	771,415	246	0.73	(0.60–0.89)
Exclusive P2 use	305,419	108	0.75	(0.59–0.96)
Duration of P2 use				
≤ 1 year	189,539	84	1.06	(0.81–1.38)
> 1–5 years	343,216	111	0.73	(0.57–0.93)
> 5 years	189,403	37	0.43	(0.30–0.61)
Continuous, in years^b^			0.90	(0.86–0.95)
Continuous, in years, adjusted for continuous duration of P1^b^			0.90	(0.86–0.94)
*p*-trend^c^				< 0.0001
*p*-trend^b^				< 0.0001
*p*-het P1 versus P2^d^				0.03

^a^Adjusted for age (months), calendar period, BMI (kg/m^2^, continuous), number of full-term pregnancies (continuous), smoking status (never/past/current), menopausal status (premenopausal/postmenopausal/perimenopausal or unknown), use of HT (never/ever), diagnosis of PCOS (yes/no), laparoscopically confirmed endometriosis (yes/no), age of menarche (years), and use of IUD/diaphragm (yes/no)

^b^Calculation including ever OC users only

^c^Calculation including never OC users

^d^Calculation using duration trends for ever OC users only

Cross-classified cumulative average doses and potencies of estrogen and progestin, in comparison to never OC use, were generally associated with lower endometrial cancer risk except for estrogen dose ≥ and progestin dose < median (1.26 [0.78–2.03]; Table 5). Among ever OC users, this category was associated with higher risk (1.76 [1.09–2.84]) relative to both average doses < median; associations were robust to adjustment for total duration of OC use. Results were similar for estrogen and progestin dose and potency residuals (data not shown).

**Table 5:** Multivariable-adjusted^a^ hazard ratios (HR) and 95% confidence intervals for endometrial cancer in relation to estrogen (E) and progestin (P) average dose and potency in the Nurses’ Health Study II (1989–2017)

	Person-years	Cases	Including never OC users		Restricted to ever OC users	
			HR	95% CI	HR	95% CI
E average dose						
Never OC use	344,113	175	1.00	(ref.)	–	–
E dose < median	1,363,352	439	0.77	(0.64–0.92)	1.00	(ref.)
E dose ≥ median	211,119	113	0.80	(0.62–1.02)	1.03	(0.83–1.28)
P average dose						
Never OC use	344,113	175	1.00	(ref.)	–	–
P dose < median	817,980	246	0.75	(0.61–0.91)	1.00	(ref.)
P dose ≥ median	764,521	312	0.80	(0.66–0.97)	1.07	(0.90–1.26)
E and P average dose						
Never OC use	344,113	175	1.00	(ref.)	–	–
Both E and P doses < median	788,189	221	0.71	(0.58–0.87)	1.00	(ref.)
E dose < and P dose ≥ median	575,164	218	0.83	(0.68–1.02)	1.17	(0.97–1.41)
E dose ≥ and P dose < median	23,219	19	1.26	(0.78–2.03)	1.76	(1.09–2.84)
Both E and P doses ≥ median	187,900	94	0.74	(0.57–0.96)	1.04	(0.81–1.34)
*p*-het				0.03		0.01
E average potency						
Never OC use	344,113	175	1.00	(ref.)	–	–
E potency < median	744,667	223	0.76	(0.62–0.94)	1.00	(ref.)
E potency ≥ median	700,808	258	0.74	(0.61–0.90)	0.97	(0.81–1.16)
P average potency						
Never OC use	344,113	175	1.00	(ref.)	–	–
P potency < median	887,756	242	0.75	(0.61–0.91)	1.00	(ref.)
P potency ≥ median	565,585	245	0.77	(0.63–0.94)	1.03	(0.85–1.23)
E and P average potency						
Never OC use	344,113	175	1.00	(ref.)	–	–
Both E and P potencies < median	519,934	148	0.78	(0.62–0.97)	1.00	(ref.)
E potency < and P potency ≥ median	224,904	75	0.74	(0.56–0.98)	0.96	(0.72–1.27)
E potency ≥ and P potency < median	361,709	88	0.68	(0.52–0.88)	0.87	(0.67–1.13)
Both E and P potencies ≥ median	339,099	170	0.78	(0.63–0.97)	1.01	(0.80–1.27)
*p*-het				0.26		0.95

^a^Adjusted for age (months), calendar period, BMI (kg/m^2^, continuous), number of full-term pregnancies (continuous), smoking status (never/past/current), menopausal status (premenopausal/postmenopausal/perimenopausal or unknown), use of HT (never/ever), diagnosis of PCOS (yes/no), laparoscopically confirmed endometriosis (yes/no), age of menarche (years), and use of IUD/diaphragm (yes/no).

### Sensitivity and subgroup analyses

We observed no significant deviations from linearity in the associations when exposures were modelled continuously in years (*p* ≥ 0.16). Results from analyses with a 2-year lag (supplementary table S6) and those restricted to cases confirmed by medical record (supplementary table S7) were similar to the overall results.

There was no statistically significant heterogeneity by age, BMI, menopausal status, HT use, or smoking status (*p*-het ≥ 0.22; data not shown).

### Analyses by tumor invasiveness

The association between short-term OC use (≤ 1 year) and risk versus never OC use for invasive carcinomas (0.77 [0.52–1.15]) was significantly different than the association for non-invasive carcinomas (1.82 [1.07–3.10]; *p*-het = 0.007; supplementary table S8). Associations for non-invasive carcinoma were similar after excluding participants with diagnoses of PCOS or endometriosis (1.75 [0.96–3.16]), and in “lagged” analysis (1.65 [0.97–2.81]). In analyses stratified by BMI, short-term use was only associated with risk of non-invasive carcinoma among women with BMI ≥ 30 kg/m^2^ (2.46 [1.18–5.10]); this finding was robust to exclusion of women diagnosed with PCOS or endometriosis and in “lagged” analysis (data not shown).

No significant heterogeneity by invasiveness was observed for the other duration categories or for duration of OC use by estrogen and progestin types, doses, and potencies (*p*-het ≥ 0.21; data not shown).

## Discussion

OC use was associated with a significantly lower risk of endometrial cancer relative to never OC use in this cohort study with up to 28 years of prospective follow-up of premenopausal women through postmenopause. Our findings of inverse associations are consistent with results from older birth cohorts (e.g., born before 1949) [15–17] and with the meta-analysis by the Collaborative Group on Epidemiological Studies on Endometrial Cancer that primarily included studies with women from earlier birth cohorts, who were more likely to have been exposed to high-dose OC formulations (43% of women with mid-calendar-year of OC use in the 1960s, 5% with mid-calendar-year of use in the 1980s) [14]. Further, longer durations of OC formulations including EE and P2 were more strongly inversely associated with risk than older formulations including ME and P1.

The inverse association between duration of OC use and endometrial cancer risk found in our study was qualitatively similar to that reported in other cohorts and a meta-analysis [14, 15, 18, 31]. Results from the meta-analysis conducted by the Collaborative Group on Epidemiological Studies on Endometrial Cancer show that the absolute risk of endometrial cancer in women prior to age 75 years was approximately halved with longer duration of OC use, from 2.3% in never OC users to 1.3 and 1.0% with 10 and 15 years of use, respectively [14]. Previous studies have described a lower risk that persisted for more than 15–20 years after cessation of use [14, 15, 17, 32, 33], and our results are in line with those reporting that the effect of duration of use is evident regardless of time since last use [14, 34, 35]. Recent analyses from the Danish Sex Hormone Register Study, a younger birth cohort, found substantial risk reductions associated with current and recent use of contemporary combined hormonal contraceptives [19]; however, this study was limited by relatively short follow-up (to age 50).

Endogenous and exogenous estrogens prompt mitotic activity in the endometrium. In women not taking OCs the follicular phase of the menstrual cycle is marked by a rise in estradiol and estrone concentrations, not counterbalanced by progesterone, promoting endometrial cell proliferation. Minimal mitotic activity of these cells is observed in women using combined OCs [31, 36]. The lower endometrial proliferation in OC users results from the concomitant administration of progestin with the estrogen, leading to down-regulation of estrogen receptors and increased metabolic inactivation of estradiol [31, 37–39]. Compared to non-users, current OC users have lower serum estradiol, which is attributed to the negative feedback promoted by the exogenous hormones at the hypothalamic-hypophyseal level [40–42], as well as higher serum levels of sex-hormone-binding globulin (SHBG), which has its production stimulated by EE [43]. The Endogenous Hormones and Breast Cancer Collaborative Group reported that past OC users have slightly lower serum estradiol and estrone concentrations before menopause [44]. Higher circulating concentrations of estrone and estradiol are associated with increased endometrial cancer risk in postmenopausal women, even after adjustment for BMI [45–48]; the mechanisms underlying the long-term lower endometrial cancer risk in past OC users are not fully elucidated.

We observed significant heterogeneity in the associations between longer duration of use of the different estrogen (ME vs. EE) and progestin (P1 vs. P2) types investigated and endometrial cancer risk. Longer durations of use of EE and P2 appeared to be more strongly associated with lower risk than long durations of use of ME and P1 (e.g., 48% lower risk with > 5 years of EE use versus 34% with ME; 57% lower risk with > 5 years of P2 use versus 38% with P1). Because all ME-based OCs used by participants were combined with P1, we were unable to assess whether the estrogen or progestin component was driving this heterogeneity. The Danish Sex Hormone Register Study’s publication reported little to no variation on endometrial cancer risk by formulation of combined hormonal contraceptive in current and recent users [19]; this study however focused on women younger than 50, when the occurrence of endometrial cancer is uncommon. Compared to never OC users, we observed lower risk regardless of cumulative average doses and potencies of estrogen and progestin, except with doses of estrogen ≥ and progestin <  the median. This is potentially related to the effect of estrogen partially unopposed by progestin, an accepted mechanism in endometrial carcinogenesis. Previous case-control studies showed no significant associations with estrogen and progestin doses and potencies, though sample sizes were limited in the subgroups [34, 35, 49, 50].

We observed no heterogeneity in associations in the overall results by BMI, contrasting with an analysis from the prospective NIH-AARP Diet and Health Study reporting that longer OC duration was associated with lower risk among overweight and obese, but not normal weight, women [18]. Findings from the Collaborative Group on Epidemiological Studies on Endometrial Cancer’s meta-analysis found no variation in the risk associations by BMI, when considering ever OC users only [14].

The natural history of endometrial cancers usually follows a sequence of benign proliferative disorder, benign hyperplasia, atypical hyperplasia, in situ/non-invasive neoplasia, to then invasive carcinoma [51]. Higher risk of non-invasive endometrial cancer was present in short-term OC users (≤ 1 year of use), with significant heterogeneity between invasive and non-invasive tumors, particularly for obese women. Associations were similar in sensitivity analyses including “lagged” analyses, suggesting that the short-term use of OCs by these women was not related to possible complaints linked to the neoplasm. Higher risk of ovarian cancer among women who used OCs for a very short duration [≤ 6 months of use, 1.82 (1.13–2.93) vs. never OC use)] was reported previously in the NHSII [23]. Given the commonality in these findings with short-term use, this association merits further exploration. In contrast with the present study’s findings, however, the use of more recent OC formulations showed no association with ovarian cancer risk [23].

Our study’s limitations include a modest number of P3 and P4 users, preventing analyses of the associations between these progestins and endometrial cancer risk. P2 exposure remains relevant as these are still among the most commonly prescribed progestins in OC formulations. The predominantly used estrogens to date were assessed (mestranol, ethinyl estradiol); estradiol valerate in OCs has been more recently introduced (e.g., U.S.: 2010), and was not evaluated. Due to a limited number of non-endometrioid cases, we were unable to assess heterogeneity by histopathological subtypes or between types I and II endometrial cancers, though a recent study found no heterogeneity between ever OC use and endometrial cancer risk by type [52]. This study has many strengths. The 28-year follow-up of this younger birth cohort, 99% of whom were premenopausal at recruitment and with detailed and updated OC use information, allowed a thorough analysis of the association of more recent OC formulations with endometrial cancer risk.

In conclusion, OC use was associated with lower endometrial cancer risk in this study, with strongest associations with EE and P2, components still commonly present in contemporary formulations. Longer duration of OC use appears to provide long-term lower risk, largely independent of time since last use and, overall, of BMI. Studies to identify subgroups of women who may benefit from longer-term OC use for primary prevention of endometrial cancer, weighing the potential risks and benefits, are warranted. Further, future studies are needed on more recently introduced progestins, and in the context of changing endometrial cancer risk profiles due to changes in contraceptive preferences (e.g., toward intrauterine devices) and high prevalence of overweight and obesity.

## Electronic Supplementary Material

Below is the link to the electronic supplementary material.

Supplementary file1 (DOCX 60 kb)

## Data Availability

Information on the procedures to obtain and access data from the Nurses’ Health Studies and Health Professionals Follow-up Study is described at https://www.nurseshealthstudy.org/researchers.
