# Supplementary material for: Oral contraceptive use by formulation and endometrial cancer risk among women born in 1947–1964: The Nurses’ Health Study II, a prospective cohort study
Source: Eur J Epidemiol. 2020 Dec 17;36(8):827–39. doi: 10.1007/s10654-020-00705-5 (PMC8416825; doi:10.1007/s10654-020-00705-5)
Supplement: Supplementary file 1 — Supplementary file1 (DOCX 60 kb) [file 10654_2020_705_MOESM1_ESM.docx]

| **Table of contents** | |
| --- | --- |
| **Page 2** | Table S1 Age-adjusted characteristics of ever OC users by ever use of mestranol (ME), ethinyl estradiol (EE), first- (P1) and second-generation (P2) progestins at study baseline in 1989: Nurses' Health Study II |
| **Page 4** | Table S2 Age-adjusted characteristics of ever OC users by ever use of mestranol (ME), ethinyl estradiol (EE), first- (P1) and second-generation (P2) progestins at last questionnaire (2015) preceding the end of the study follow-up in 2017: Nurses' Health Study II |
| **Page 6** | Table S3 Characteristics of oral contraceptive use among ever OC users in the study population in 1989: Nurses’ Health Study II (n=88,433) |
| **Page 8** | Table S4 Cross-tabulation of the proportion of ever users of mestranol (ME), ethinyl estradiol (EE), first- (P1) and second-generation (P2) progestins at last questionnaire (2015) preceding the end of the study follow-up in 2017: Nurses' Health Study II |
| **Page 9** | Table S5 Multivariable-adjusted hazard ratios (HR) and 95% confidence intervals for endometrial cancer in relation to duration of OC use (continuous, in years) by time since last OC use, and time since last OC use (continuous, in years) by duration of OC use among ever OC users in the Nurses’ Health Study II (1989-2017) |
| **Page 10** | Table S6 Multivariable-adjusted hazard ratios (HR) and 95% confidence intervals for endometrial cancer in relation to OC use, with OC use updated 2 years prior to the end of follow-up, in the Nurses’ Health Study II (1989-2017) |
| **Page 11** | Table S7 Multivariable-adjusted hazard ratios (HR) and 95% confidence intervals for endometrial cancer in relation to association between ever OC use, duration of OC use and time since last OC use in the Nurses’ Health Study II (1989-2017) including cases confirmed by medical record only |
| **Page 12** | Table S8 Multivariable-adjusted hazard ratios (HR) and 95% confidence intervals for non-invasive and invasive endometrial carcinomas in relation to ever OC use, duration of OC use and time since last OC use in the Nurses’ Health Study II (1989-2017) |

**Supplementary Materials**

| **Table S1 Age-adjusted characteristics of ever OC users by ever use of mestranol (ME), ethinyl estradiol (EE), first- (P1) and second-generation (P2) progestins at study baseline in 1989: Nurses' Health Study II** | | | | | | | | |
| --- | --- | --- | --- | --- | --- | --- | --- | --- |
|  | **ME use** | | **EE use** | | **P1 use** | | **P2 use** | |
|  | Never ME use (n=50,668) | Ever ME use (n=37,765) | Never EE use (n=29,817) | Ever EE use (n=58,616) | Never P1 use (n=25,136) | Ever P1 use (n=63,297) | Never P2 use (n=55,653) | Ever P2 use (n=32,780) |
| Age, years^*^ | 33.0 [24-44] | 36.0 [24-44] | 37.0 [24-44] | 33.0 [24-44] | 35.0 [24-44] | 34.0 [24-44] | 35.0 [24-44] | 34.0 [24-44] |
| Age at menarche | 12 [9-17] | 12.0 [9-17] | 12.0 [9-17] | 12.0 [9-17] | 12.0 [9-17] | 12.0 [9-17] | 12.0 [9-17] | 12.0 [9-17] |
| BMI^a^, categorical |  |  |  |  |  |  |  |  |
| - BMI<25kg/m^2^, % | 72.0 | 69.9 | 70.1 | 71.6 | 70.8 | 71.3 | 71.3 | 70.9 |
| - BMI 25-<30kg/m^2^, % | 17.7 | 18.9 | 18.5 | 18.1 | 18.2 | 18.2 | 18.0 | 18.5 |
| - BMI≥30kg/m^2^, % | 10.2 | 11.2 | 11.3 | 10.3 | 11.0 | 10.5 | 10.6 | 10.6 |
| Ever parous, % | 69.6 | 77.1 | 80.3 | 69.2 | 74.6 | 71.9 | 73.5 | 71.1 |
| Number of full-term pregnancies (among parous women) | 2.0 [1-15] | 2.0 [1-11] | 2.0 [1-15] | 2.0 [1-10] | 2.0 [1-15] | 2.0 [1-11] | 2.0 [1-15] | 2.0 [1-10] |
| Breastfeeding, in months (among parous women who ever breastfed) | 9.0 [1.5-95] | 10.0 [1.5-91] | 10.0 [1.5-95] | 9.0 [1.5-91] | 9.0 [1.5-95] | 9.0 [1.5-91] | 9.5 [1.5-95] | 9.0 [1.5-91] |
| Menopausal status |  |  |  |  |  |  |  |  |
| - Premenopausal, % | 99.6 | 99.5 | 99.5 | 99.6 | 99.6 | 99.6 | 99.6 | 99.6 |
| - Postmenopausal, % | 0.2 | 0.3 | 0.3 | 0.2 | 0.2 | 0.3 | 0.3 | 0.2 |
| - Missing, % | 0.2 | 0.2 | 0.2 | 0.2 | 0.2 | 0.2 | 0.2 | 0.2 |
| Postmenopausal HT^b^ use, % | 0.2 | 0.2 | 0.2 | 0.2 | 0.2 | 0.2 | 0.2 | 0.2 |
| Ever use of other contraceptive methods |  |  |  |  |  |  |  |  |
| - IUD^c^ or diaphragm, % | 12.5 | 13.0 | 14.2 | 12.1 | 12.7 | 12.7 | 13.2 | 11.8 |
| - Male contraception, % | 30.1 | 31.8 | 33.0 | 29.9 | 31.3 | 30.6 | 31.3 | 30.0 |
| - Tubal ligation, % | 14.8 | 20.6 | 22.3 | 14.9 | 18.2 | 16.8 | 17.6 | 16.5 |
| - Other, % | 15.4 | 15.0 | 15.2 | 15.3 | 14.7 | 15.5 | 15.8 | 14.4 |
| Diagnosis of endometriosis^d^, % | 3.3 | 3.6 | 3.2 | 3.5 | 3.6 | 3.3 | 2.9 | 4.2 |
| Diagnosis of PCOS^e^, % | 5.4 | 5.9 | 5.7 | 5.6 | 5.4 | 5.7 | 5.4 | 5.9 |
| Ever migraines, % | 14.4 | 16.0 | 15.3 | 15.0 | 14.8 | 15.2 | 14.4 | 16.1 |
| Arterial hypertension, % | 4.8 | 5.7 | 5.7 | 5.0 | 5.2 | 5.2 | 5.1 | 5.3 |
| Diabetes mellitus, % | 0.6 | 0.7 | 0.8 | 0.6 | 0.8 | 0.6 | 0.7 | 0.6 |
| Smoking status |  |  |  |  |  |  |  |  |
| - Never smoker, % | 66.0 | 59.1 | 60.7 | 64.3 | 63.4 | 63.0 | 63.8 | 62.2 |
| - Past smoker, % | 21.2 | 25.1 | 24.4 | 22.1 | 22.8 | 22.8 | 22.5 | 23.3 |
| - Current smoker, % | 12.8 | 15.8 | 15.0 | 13.6 | 13.8 | 14.2 | 13.7 | 14.5 |
| Values are medians and ranges or percentages and are standardized to the age distribution of the study population.  Values of polytomous variables may not sum to 100% due to rounding.  ^a^ Body mass index.  ^b^ Hormone therapy.  ^c^ Intrauterine device.  ^d^ Laparoscopically confirmed endometriosis.  ^e^ Polycystic Ovaries Syndrome.  ^*^ Value is not age adjusted. | | | | | | | | |

| **Table S2 Age-adjusted characteristics of ever OC users by ever use of mestranol (ME), ethinyl estradiol (EE), first- (P1) and second-generation (P2) progestins at last questionnaire (2015) preceding the end of the study follow-up in 2017: Nurses' Health Study II** | | | | | | | | |
| --- | --- | --- | --- | --- | --- | --- | --- | --- |
|  | **ME use** | | **EE use** | | **P1 use** | | **P2 use** | |
|  | Never ME use (n=32,417) | Ever ME use (n=19,964) | Never EE use (n=19,244) | Ever EE use (n=33,137) | Never P1 use (n=17,950) | Ever P1 use (n=34,431) | Never P2 use (n=33,049) | Ever P2 use (n=19,332) |
| Age, years^*^ | 59.0 [50-71] | 62.0 [50-71] | 62.0 [50-71] | 60.0 [50-71] | 60.0 [50-71] | 60 [50-71] | 61.0 [50-71] | 60 [50-71] |
| BMI^a^, categorical |  |  |  |  |  |  |  |  |
| - BMI<25kg/m^2^, % | 37.1 | 36.3 | 36.9 | 36.8 | 36.6 | 37.0 | 37.0 | 36.6 |
| - BMI 25-<30kg/m^2^, % | 38.3 | 37.7 | 39.1 | 37.6 | 39.0 | 37.6 | 38.2 | 37.9 |
| - BMI≥30kg/m^2^, % | 24.6 | 26.0 | 24.0 | 25.6 | 24.3 | 25.5 | 24.8 | 25.6 |
| Ever parous, % | 83.3 | 84.9 | 84.6 | 83.1 | 83.3 | 84.2 | 84.3 | 83.0 |
| Number of full-term pregnancies (among parous women) | 2.0 [1-15] | 2.0 [1-11] | 2.0 [1-15] | 2.0 [1-11] | 2.0 [1-15] | 2.0 [1-11] | 2.0 [1-15] | 2.0 [1-11] |
| Breastfeeding, in months (among parous women who ever breastfed) | 13.5 [1.5-114] | 14.0 [1.5-110] | 13.5 [1.5-106] | 14.0 [1.5-114] | 12.0 [1.5-106] | 14.0 [1.5-114] | 14.0 [1.5-114] | 13.5 [1.5-91] |
| Menopausal status |  |  |  |  |  |  |  |  |
| - Premenopausal, % | 4.0 | 3.3 | 3.4 | 4.0 | 3.6 | 4.0 | 3.9 | 3.8 |
| - Postmenopausal, % | 86.0 | 88.0 | 86.1 | 86.7 | 86.0 | 86.8 | 86.4 | 86.7 |
| - Dubious/missing, % | 10.1 | 8.7 | 10.4 | 9.3 | 10.4 | 9.2 | 9.8 | 9.4 |
| Postmenopausal HT^b^ use, % | 31.2 | 33.8 | 31.6 | 32.5 | 31.1 | 32.7 | 31.4 | 33.4 |
| Ever use of other contraceptive methods |  |  |  |  |  |  |  |  |
| - IUD^c^ or diaphragm, % | 21.0 | 20.2 | 20.7 | 20.6 | 20.5 | 20.7 | 20.9 | 20.1 |
| - Male contraception, % | 59.7 | 58.0 | 57.6 | 59.9 | 58.7 | 59.4 | 58.8 | 59.7 |
| - Tubal ligation, % | 26.0 | 30.5 | 27.9 | 27.3 | 26.2 | 28.3 | 27.3 | 28.0 |
| - Other, % | 34.8 | 32.8 | 33.0 | 34.7 | 34.1 | 34.0 | 34.1 | 34.0 |
| Diagnosis of endometriosis^d^, % | 6.7 | 6.4 | 6.1 | 6.9 | 6.8 | 6.4 | 6.0 | 7.3 |
| Diagnosis of PCOS^e^, % | 7.7 | 7.8 | 8.1 | 7.5 | 7.9 | 7.6 | 7.6 | 7.8 |
| Ever migraines, % | 44.4 | 44.8 | 44.2 | 45.0 | 44.0 | 45.0 | 44.1 | 45.6 |
| Arterial hypertension, % | 4.1 | 4.6 | 4.2 | 4.4 | 4.0 | 4.5 | 4.3 | 4.4 |
| Diabetes mellitus, % | 0.5 | 0.5 | 0.6 | 0.5 | 0.6 | 0.5 | 0.5 | 0.5 |
| Smoking status |  |  |  |  |  |  |  |  |
| - Never smoker, % | 64.9 | 59.4 | 63.0 | 63.2 | 64.2 | -62.5 | 63.9 | 61.8 |
| - Past smoker, % | 29.7 | 34.2 | 31.2 | 31.1 | 30.3 | 31.6 | 30.6 | 32.2 |
| - Current smoker, % | 5.4 | 6.5 | 5.8 | 5.7 | 5.5 | 5.9 | 5.6 | 6.1 |
| Values are medians and ranges or percentages and are standardized to the age distribution of the study population.  Values of polytomous variables may not sum to 100% due to rounding.  ^a^ Body mass index.  ^b^ Hormone therapy.  ^c^ Intrauterine device.  ^d^ Laparoscopically confirmed endometriosis.  ^e^ Polycystic Ovaries Syndrome.  ^*^ Value is not age adjusted. | | | | | | | | |

| **Table S3 Characteristics of oral contraceptive use among ever OC users in the study population in 1989: Nurses’ Health Study II (n=88,433^a^)** | |
| --- | --- |
| **OC use status, among ever users** | |
| - Current OC user, % | 14.9 |
| - Past OC user, % | 85.1 |
| **Estrogen**^b,c^ | |
| - Cumulative dose | 50,000 (913.0-600,000) |
| - Cumulative potency | 36,981 (730.5-1,132,731) |
| - Cumulative average dose | 1,000 (23.0-3,000) |
| - Cumulative average potency | 795.7 (22.8-9,999) |
| **Progestin**^d,e^ | |
| - Cumulative dose | 822.0 (4.0-50,000) |
| - Cumulative potency | 1,095 (18.3-45,994) |
| - Cumulative average dose | 21.0 (2.0-225) |
| - Cumulative average potency | 22.8 (5.4-225.5) |
| **Number of OC brands ever used** | |
| - 1 brand used, % | 48.6 |
| - 2 brands used, % | 25.7 |
| - 3 brands used, % | 8.8 |
| - 4 or more brands used, % | 3.7 |
| - Used OCs but unknown number of brands, % | 13.3 |
| **Estrogen type used** | |
| Mestranol (ME) |  |
| - Ever use, % | 42.7 |
| - Exclusive use, % | 23.1 |
| - Duration of use, continuous in years^f^ | 3.0 (0.2-22.0) |
| Ethinyl estradiol (EE) |  |
| - Ever use, % | 66.3 |
| - Exclusive use of ethinyl estradiol, % | 46.7 |
| - Duration of use, continuous in years^g^ | 3.0 (0.2-25.0) |
| Missing estrogen type, % | 10.5 |
| **Progestin type used** | |
| First-generation progestin (P1)^h^ |  |
| - Ever use, % | 71.6 |
| - Exclusive use, % | 52.5 |
| - Duration of use, continuous in years^i^ | 3.0 (0.2-25.0) |
| Second-generation progestin (P2)^j^ |  |
| - Ever use, % | 37.1 |
| - Exclusive use, % | 18.0 |
| - Duration of use, continuous in years^k^ | 2.5 (0.2-24.3) |
| Missing progestin type, % | 10.5 |
| Values are medians and range or percentages and are standardized to the age distribution of the study population.  Values of polytomous variables may not sum to 100% due to rounding.  ^a^Never OC users, n=18,636 (17.4% of the total population)  ^b^N=9,383 (10.6%) ever OC users missing cumulative estrogen dose and N=15,271 (17.2%) missing cumulative estrogen potency.  ^c^Units are as follows: cumulative estrogen dose: µg; cumulative estrogen potency: µg ethinyl estradiol equivalents; cumulative average estrogen dose: µg per month; cumulative average estrogen potency: µg ethinyl estradiol equivalents per month.  ^d^N=9,251 (10.4%) ever OC users missing cumulative progestin dose and N=15,138 (17.1%) missing cumulative progestin potency.  ^e^Units are as follows: cumulative progestin dose: mg; cumulative progestin potency: mg norethindrone equivalents; cumulative average progestin dose: mg per month; cumulative average progestin potency: mg norethindrone equivalents per month.  ^f^Among ever users of mestranol.  ^g^Among ever users of ethinyl estradiol.  ^h^First-generation progestin includes norethindrone, norethynodrel, norethindrone acetate, ethynodiol diacetate, medroxyprogesterone acetate, and chlormadinone acetate.  ^i^Among ever users of first-generation progestins.  ^j^Second-generation progestin includes levonorgestrel and norgestrel.  ^k^Among ever users of second-generation progestins. | |

| **Table S4 Cross-tabulation of the proportion of ever users of mestranol (ME), ethinyl estradiol (EE), first- (P1) and second-generation (P2) progestins at last questionnaire (2015) preceding the end of the study follow-up in 2017: Nurses' Health Study II** | | | | | |
| --- | --- | --- | --- | --- | --- |
| **Of women using hormone below, proportion who were ever users of:** | **ME** | **EE** | **P1** | **P2** | n |
| **Ever ME** |  | 55% | 100% | 29% | *21,180* |
| **Ever EE** | 32% |  | 76% | 57% | *36,871* |
| **Ever P1** | 56% | 74% |  | 34% | *37,723* |
| **Ever P2** | 29% | 100% | 60% |  | *21,206* |

| **Table S5 Multivariable-adjusted^a^ hazard ratios (HR) and 95% confidence intervals for endometrial cancer in relation to duration of OC use (continuous, in years) by time since last OC use, and time since last OC use (continuous, in years) by duration of OC use among ever OC users in the Nurses’ Health Study II (1989-2017)** | | | | | | |
| --- | --- | --- | --- | --- | --- | --- |
|  | **Person-years** | **Cases** | Adjusted for age and calendar period | | Multivariable-adjusted^a^ | |
|  |  |  | **HR** | **95% CI** | **HR** | **95% CI** |
| **Associations for duration of OC use (continuous, years) in categories of time since last OC use** | | | | | | |
| >15yrs | 858,819 | 490 | 0.96 | (0.94-0.99) | 0.95 | (0.93-0.98) |
| >10-15yrs | 291,183 | 69 | 0.89 | (0.84-0.95) | 0.90 | (0.85-0.96) |
| >5-10yrs | 266,396 | 52 | 0.93 | (0.88-0.99) | 0.92 | (0.87-0.97) |
| Current use/≤5yrs since last use | 489,738 | 62 | 0.94 | (0.90-0.99) | 0.94 | (0.90-0.98) |
| **Associations for time since last OC use (continuous, years) in categories of duration of OC use** | | | | | | |
| ≤1y | 324,673 | 148 | 0.99 | (0.97-1.01) | 0.99 | (0.97-1.02) |
| >1-5yrs | 732,052 | 318 | 1.00 | (0.99-1.01) | 1.00 | (0.98-1.01) |
| >5-10yrs | 456,050 | 137 | 1.02 | (1.00-1.04) | 1.01 | (0.99-1.04) |
| >10yrs | 212,064 | 52 | 1.03 | (0.98-1.07) | 1.02 | (0.98-1.07) |
| ^a^Adjusted for age (months), calendar period, BMI (kg/m^2^, continuous), number of full-term pregnancies (continuous), smoking status (never/past/current), menopausal status (premenopausal or dubious/postmenopausal), use of HT (never/ever), diagnosis of PCOS (yes/no), laparoscopically confirmed endometriosis (yes/no), age of menarche (years), and use of IUD/diaphragm (yes/no). | | | | | | |

| **Table S6 Multivariable-adjusted^a^ hazard ratios (HR) and 95% confidence intervals for endometrial cancer in relation to OC use, with OC use updated 2 years prior to the end of follow-up, in the Nurses’ Health Study II (1989-2017)** | | | | |
| --- | --- | --- | --- | --- |
|  | **Person-years** | **Cases** | **HR** | **95% CI** |
| **OC use** | | | | |
| Never use | 319,950 | 171 | 1.00 | (ref.) |
| Ever use | 1,803,997 | 677 | 0.78 | (0.66-0.93) |
| **Duration of OC use** | | | | |
| Never use | 319,950 | 171 | 1.00 | (ref.) |
| ≤1y | 308,899 | 149 | 0.97 | (0.78-1.22) |
| >1-5yrs | 724,157 | 322 | 0.91 | (0.76-1.10) |
| >5-10yrs | 488,601 | 143 | 0.65 | (0.51-0.81) |
| >10yrs | 282,342 | 63 | 0.46 | (0.34-0.62) |
| Continuous duration of use, in years^b^ |  |  | 0.94 | (0.92-0.96) |
| *p*-trend^c^ |  |  |  | <.0001 |
| *p*-trend^b^ |  |  |  | <.0001 |
| **Duration of OC use and time since last OC use**^d^ | | | | |
| Never use | 319,950 | 171 | 1.00 | (ref.) |
| ≤1y of use | 308,897 | 149 | 0.97 | (0.78-1.21) |
| >1-5yrs of use and ≤10yrs since last use | 187,570 | 42 | 0.89 | (0.63-1.25) |
| >1-5yrs of use and >10yrs since last use | 485,777 | 271 | 0.92 | (0.76-1.12) |
| >5yrs of use and ≤10yrs since last use | 302,842 | 46 | 0.45 | (0.32-0.62) |
| >5yrs of use and >10yrs since last use | 303,303 | 139 | 0.64 | (0.51-0.80) |
| ^a^Adjusted for age (months), calendar period, BMI (kg/m^2^, continuous), number of full-term pregnancies (continuous), smoking status (never/past/current), menopausal status (premenopausal/postmenopausal/perimenopausal or unknown), use of HT (never/ever), diagnosis of PCOS (yes/no), laparoscopically confirmed endometriosis (yes/no), age of menarche (years), and use of IUD/diaphragm (yes/no).  ^b^Calculation including ever OC users only.  ^c^Calculation including never OC users.  ^d^Current OC users were excluded from this analysis. | | | | |

| **Table S7 Multivariable-adjusted^a^ hazard ratios (HR) and 95% confidence intervals for endometrial cancer in relation to association between ever OC use, duration of OC use and time since last OC use in the Nurses’ Health Study II (1989-2017) including cases confirmed by medical record only** | | | | |
| --- | --- | --- | --- | --- |
|  | **Person-years** | **Cases** | **HR** | **95% CI** |
| **OC use** | | | | |
| Never use | 344,113 | 137 | 1.00 | (ref.) |
| Ever use | 1,957,361 | 548 | 0.79 | (0.65-0.95) |
| **Duration of OC use** | | | | |
| Never use | 344,113 | 137 | 1.00 | (ref.) |
| ≤1y | 334,666 | 123 | 0.98 | (0.77-1.26) |
| >1-5yrs | 783,484 | 264 | 0.94 | (0.76-1.15) |
| >5-10yrs | 529,236 | 119 | 0.67 | (0.52-0.86) |
| >10yrs | 309,975 | 42 | 0.38 | (0.26-0.53) |
| Continuous duration of use, in years^b^ |  |  | 0.93 | (0.91-0.95) |
| *p*-trend^c^ |  |  |  | <.0001 |
| *p*-trend^b^ |  |  |  | <.0001 |
| **Duration of OC use and time since last use OC use**^d^ | | | | |
| Never use | 344,113 | 137 | 1.00 | (ref.) |
| ≤1y of use | 334,666 | 123 | 0.98 | (0.77-1.26) |
| >1-5yrs of use and ≤10yrs since last use | 197,255 | 28 | 0.81 | (0.54-1.23) |
| >1-5yrs of use and >10yrs since last use | 534,797 | 230 | 0.96 | (0.77-1.20) |
| >5yrs of use and ≤10yrs since last use | 324,053 | 29 | 0.38 | (0.25-0.58) |
| >5yrs of use and >10yrs since last use | 344,061 | 121 | 0.66 | (0.51-0.84) |
| ^a^Adjusted for age (months), calendar period, BMI (kg/m^2^, continuous), number of full-term pregnancies (continuous), smoking status (never/past/current), menopausal status (premenopausal/postmenopausal/perimenopausal or unknown), use of HT (never/ever), diagnosis of PCOS (yes/no), laparoscopically confirmed endometriosis (yes/no), age of menarche (years), and use of IUD/diaphragm (yes/no).  ^b^Calculation including ever OC users only.  ^c^Calculation including never OC users.  ^d^Current OC users were excluded from this analysis. | | | | |

| **Table S8 Multivariable-adjusted^a^ hazard ratios (HR) and 95% confidence intervals for non-invasive and invasive endometrial carcinomas in relation to ever OC use, duration of OC use and time since last OC use in the Nurses’ Health Study II (1989-2017)** | | | | | | | | |
| --- | --- | --- | --- | --- | --- | --- | --- | --- |
|  | **Person-years** | **Non-invasive carcinomas** | | | **Invasive carcinomas** | | | *p*-het by subtype |
|  |  | **Cases** | **HR** | **95% CI** | **Cases** | **HR** | **95% CI** |  |
| **OC use** | | | | | | | | |
| Never use | 344,113 | 23 | 1.00 | (ref.) | 60 | 1.00 | (ref.) |  |
| Ever use | 1,957,361 | 106 | 0.97 | (0.61-1.53) | 196 | 0.66 | (0.49-0.89) | 0.12 |
| **Duration of OC use** | | | | | | | | |
| Never use | 344,113 | 23 | 1.00 | (ref.) | 60 | 1.00 | (ref.) |  |
| ≤1y | 334,666 | 37 | 1.82 | (1.07-3.10) | 43 | 0.77 | (0.52-1.15) | 0.007 |
| >1-5yrs | 783,484 | 40 | 0.89 | (0.53-1.50) | 88 | 0.72 | (0.52-1.01) |  |
| >5-10yrs | 529,236 | 21 | 0.74 | (0.41-1.37) | 51 | 0.69 | (0.47-1.01) |  |
| >10yrs | 309,975 | 8 | 0.48 | (0.21-1.09) | 14 | 0.32 | (0.18-0.58) |  |
| *p*-trend^b^ |  |  |  | 0.001 |  |  | 0.0004 | 0.54 |
| *p*-trend^c^ |  |  |  | 0.0008 |  |  | 0.01 | 0.12 |
| **Duration of OC use and time since last use OC use**^d^ | | | | | | | | |
| Never use | 344,113 | 23 | 1.00 | (ref.) | 60 | 1.00 | (ref.) |  |
| ≤1y of use | 334,666 | 37 | 1.82 | (1.07-3.10) | 43 | 0.77 | (0.52-1.15) | 0.001 |
| >1-5yrs of use and ≤10yrs since last use | 197,255 | 7 | 0.97 | (0.41-2.30) | 10 | 0.57 | (0.29-1.12) |  |
| >1-5yrs of use and >10yrs since last use | 534,797 | 33 | 0.90 | (0.52-1.55) | 75 | 0.75 | (0.53-1.06) |  |
| >5yrs of use and ≤10yrs since last use | 324,053 | 11 | 0.79 | (0.38-1.65) | 5 | 0.14 | (0.06-0.36) |  |
| >5yrs of use and >10yrs since last use | 344,061 | 16 | 0.63 | (0.33-1.21) | 53 | 0.75 | (0.51-1.09) |  |
| ^a^Adjusted for age (months), calendar period, BMI (kg/m^2^, continuous), number of full-term pregnancies (continuous), smoking status (never/past/current), menopausal status (premenopausal/postmenopausal/perimenopausal or unknown), use of HT (never/ever), diagnosis of PCOS (yes/no), laparoscopically confirmed endometriosis (yes/no), age of menarche (years), and use of IUD/diaphragm (yes/no).  ^b^Calculation including never OC users.  ^c^Calculation including ever OC users only.  ^d^Current OC users were excluded from this analysis. | | | | | | | | |
